# Decoy TRAIL receptor CD264: a cell surface marker of cellular aging for human bone marrow-derived mesenchymal stem cells

**DOI:** 10.1186/s13287-017-0649-4

**Published:** 2017-09-29

**Authors:** Sean D. Madsen, Katie C. Russell, H. Alan Tucker, Julie Glowacki, Bruce A. Bunnell, Kim C. O’Connor

**Affiliations:** 1 Department of Chemical and Biomolecular Engineering, Tulane University, New Orleans, Louisiana USA; 2 Biomedical Sciences Graduate Program, Tulane University School of Medicine, New Orleans, Louisiana USA; 3 Center for Stem Cell Research and Regenerative Medicine, Tulane University School of Medicine, New Orleans, Louisiana USA; 4 Department of Orthopedic Surgery, Brigham and Women’s Hospital, Harvard Medical School, Boston, Massachusetts USA; 5 Center for Aging, Tulane University School of Medicine, New Orleans, Louisiana USA

**Keywords:** Mesenchymal stem cells, Cellular aging, Heterogeneity, Decoy TRAIL receptor, CD264

## Abstract

**Background:**

Mesenchymal stem cells (MSCs) are a mixture of progenitors that are heterogeneous in their regenerative potential. Development of MSC therapies with consistent efficacy is hindered by the absence of an immunophenotype of MSC heterogeneity. This study evaluates decoy TRAIL receptor CD264 as potentially the first surface marker to detect cellular aging in heterogeneous MSC cultures.

**Methods:**

CD264 surface expression, regenerative potential, and metrics of cellular aging were assessed in vitro for marrow MSCs from 12 donors ages 20–60 years old. Male and female donors were age matched.

Expression of CD264 was compared with that of p16, p21, and p53 during serial passage of MSCs.

**Results:**

When CD264^+^ cell content was 20% to 35%, MSC cultures from young (ages 20–40 years) and older (ages 45–60 years) donors proliferated rapidly and differentiated extensively. Older donor MSCs containing < 35% CD264^+^ cells had a small size and negligible senescence despite the donor’s advanced chronological age. Above the 35% threshold, CD264 expression inversely correlated with proliferation and differentiation potential. When CD264^+^ cell content was 75%, MSCs were enlarged and mostly senescent with severely compromised regenerative potential. There was no correlation of the older donors’ chronological age to either CD264^+^ cell content or the regenerative potential of the donor MSCs. CD264 was upregulated after p53 and had a similar expression profile to that of p21 during serial passage of MSCs. No sex-linked differences were detected in this study.

**Conclusions:**

These results suggest that CD264 is a surface marker of cellular age for MSCs, not the chronological age of the MSC donor. CD264 is first upregulated in MSCs at an intermediate stage of cellular aging and remains upregulated as aging progresses towards senescence. The strong inverse correlation of CD264^+^ cell content to the regenerative potential of MSCs has possible application to assess the therapeutic potential of patient MSCs, standardize the composition and efficacy of MSC therapies, and facilitate aging research on MSCs.

**Electronic supplementary material:**

The online version of this article (doi:10.1186/s13287-017-0649-4) contains supplementary material, which is available to authorized users.

## Background

The remarkable regenerative capacity and numerous therapeutic applications of mesenchymal stem cells (MSCs) are well documented [1, 2]; however, the underlying cellular heterogeneity of MSCs remains a major impediment to the standardization and optimization of MSC therapies for clinical use. Functional heterogeneity of MSC populations has been reported for their differentiation and proliferation potential [3, 4], as well as their trophic properties [5, 6]. Cell-to-cell variation in the regenerative properties of MSCs has been attributed to distinct in vivo phenotypes, ex vivo culturing conditions, and cell senescence [7, 8]. Differences in the composition of MSC populations among subjects can compound genotypic differences, giving rise to donor-to-donor variation in the function of MSC cultures [9]. Cellular heterogeneity in MSC therapies can cause variability in treatment outcome. For example, greater cardiac repair was observed after treating infarcted myocardium with multipotent MSC clones than with the parental culture [10]. It is infeasible at present to standardize the composition of cell populations in MSC therapies because an immunophenotype to characterize MSC heterogeneity is poorly defined [8]. Identifying surface markers for specific MSC populations is an initial step toward the production of MSC therapies with consistent efficacy.

Previous immunophenotyping studies by ourselves and others to characterize MSC heterogeneity have focused on isolating multipotent progenitors with positive selection markers such as stem cell antigen 1 [11] and neuron-glial antigen 2 [12]. In contrast to these earlier studies, our approach here is to identify an aging cell population in heterogeneous MSC cultures. Existing methods to detect senescent cells are inadequate to standardize the composition of MSC therapies; they often require cell fixation and intracellular labeling [13, 14]. Standardization of MSC composition for clinical applications necessitates use of live cells with minimal manipulation; for example, by immunolabeling surface antigens [15]. Currently, there are no known surface markers of cellular aging for MSCs or any other type of stem cell [14]. A biomarker of cellular aging would facilitate: 1) enrichment of multipotent progenitors by negative selection [16]; 2) selection of culture conditions to slow cellular aging during MSC expansion [17]; 3) potential rejuvenation of aging MSCs [18]; and 4) research into the biology of MSC aging [19].

This study examines CD264 (also known as TRAIL-R4, DcR2, TRUNDD, and TNFRSF10D) as a potential biomarker of cellular aging for MSCs. CD264 is a decoy receptor for tumor necrosis factor-related apoptosis-inducing ligand (TRAIL) [20]. A truncated intracellular death domain prevents CD264 from signaling for TRAIL-induced apoptosis [20]. Instead, this decoy receptor inhibits cell death by competing for TRAIL binding [21] and obstructing the formation of a death-inducing signaling complex [22]. A link of CD264 to senescence was first reported for oncogene-induced senescence; CD264 was upregulated in senescent premalignant tumors induced by *ras* but was downregulated in the *ras*-induced tumors that had escaped senescence and became malignant [23]. Subsequently, an increase in CD264 expression was detected upon stress-induced senescence of non-malignant smooth muscle cells [24]. In contrast, CD264 was reported not to be upregulated in association with replicative senescence [23], suggesting that this decoy receptor is not uniformly a senescence marker across different types of cells. CD264 is widely expressed in fetal and adult tissues of human origin [25, 26], and therefore may have non-senescence functions. For some cell types, expression of CD264 decreases or remains unchanged upon stress-induced senescence [27]. When taken in aggregate, these findings suggest that CD264 is not a general marker of senescence and needs to be evaluated on a case-by-case basis.

There is limited information on CD264 expression in MSCs. CD264 has been examined in the context of genetically engineered MSCs that overexpress TRAIL as a potential treatment to kill cancer cells [28]. MSCs are resistant to TRAIL-induced apoptosis [29]. Some researchers detected CD264 on the surface of bone marrow MSCs [28, 29], but another group reported that marrow MSCs are negative for CD264 surface expression [30]. A recent abstract mentions that CD264 becomes upregulated in late passage compared to early passage MSCs [31]. Our research seeks to clarify the conflicting data about CD264 expression in MSCs. Here we explore donor-to-donor variation in CD264 expression in MSC cultures and the stage of cellular aging at which CD264 becomes upregulated in MSCs. This information is essential to evaluate CD264 as potentially the first cell surface marker to detect cellular aging in heterogeneous MSC cultures.

## Methods

### MSC cultures

Primary MSCs were harvested from bone marrow [32] that was aspirated from healthy adult volunteers or discarded during orthopedic surgery after approval by the institutional review boards at Brigham and Women’s Hospital and Tulane University School of Medicine. Plastic-adherent MSCs prior to expansion are designated as passage (P)0. MSC cultures employed in this study satisfy the plastic-adherence, potency, and immunophenotype criteria for defining human MSCs specified by the International Society for Cellular Therapy [33] (Additional file 1: Table S1). All cell culture supplies herein were obtained from Life Technologies (Carlsbad, CA, USA) except where noted, and 100 U/ml penicillin and 100 μg/ml streptomycin were added to all media. For routine cultivation, MSCs were inoculated at 100 cells/cm^2^ in T flasks containing complete culture medium (CCMA) of α-MEM with 2 mM l-glutamine, supplemented with 17% fetal bovine serum (FBS) and an additional 2 mM l-glutamine [34]. Cultures were maintained in a 37 °C humidified incubator at 5% CO_2_ with complete medium exchange every 3–4 days and were subcultured at 50% confluence with 0.25% trypsin/1 mM EDTA.

### Other cultures

COLO 205 (ATCC CCL-222, Manassas, VA, USA), MDA-MB-468 (ATCC HTB-132), MCF7 (ATCC HTB-22), and HT29 (ECACC Collection, Sigma-Aldrich, St. Louis, MO, USA) human cell lines were employed as positive controls for expression of CD264, p16, p21, and p53 (Additional file 2: Figure S1 and Additional file 3: Figure S2) [30, 35–37]. These cells were cultured according to supplier’s instructions. MCF7 cells were exposed to 50 nM taxol (Sigma-Aldrich) for 36 h to upregulate p21 expression [37].

### Flow cytometry

Immunolabeling was performed on the surface of live MSCs to detect CD264 and MSC surface markers [33], as well as in fixed and permeabilized MSCs to determine the expression of intracellular cell cycle inhibitors. These antigens were detected with fluorochrome-conjugated, anti-human monoclonal antibodies relative to matched isotype controls. The antibody to detect CD264 was obtained from R&D Systems (Minneapolis, MN, USA). Additional file 1 (Table S1) lists antibodies and their suppliers for routine immunophenotyping of MSCs. Antibodies for intracellular labeling of cell cycle inhibitors p53 and p21 were acquired from Cell Signaling Technology (Danvers, MA, USA) and p16 antibodies were acquired from BD Biosciences (Franklin Lakes, NJ, USA).

MSC cultures were gently trypsinized with 0.25% trypsin/1 mM EDTA for 3 min. Trypsin was deactivated with CCMA before the cells were resuspended at 1 × 10^6^ cells/ml in phosphate-buffered saline (PBS). Antibodies were used at saturating conditions determined by titration. Cell surface labeling was achieved by incubating the cell solution with antibody for 30 min in the dark on ice. For surface labeling only, the labeled cells were washed 2× with PBS, resuspended at 5–6 × 10^5^ cells/ml PBS and kept on ice until ready to be analyzed. Intracellular labeling of MSCs for p53 and p21 was achieved using the Transcription Factor Buffer Set (BD Biosciences) according to the manufacturer’s instructions. Intracellular labeling for p16 was accomplished using a 4% formaldehyde fixation and 90% methanol (Sigma-Aldrich) permeabilization as described by Cell Signaling Technology. When co-labeling CD264 and intracellular proteins, cells were first surface labeled as described above, and then fixed and permeabilized using the Transcription Factor Buffer Set before intracellular labeling. DNA in MCF7 cells was labeled with PI/RNase staining solution (Cell Signaling Technology) after immunostaining for p21 according to the manufacturer’s instructions.

Flow cytometric analysis was performed as described by Russell et al. [12]. Labeled cells were analyzed on a Gallios flow cytometer with Kaluza software (version 1.3, Beckman Coulter, Brea, CA, USA), FACSVantage SE/DiVa flow cytometer with FACSDiVa software (version 5.0.2, BD Biosciences), or FACSAria flow cytometer with FACSDiva software (version 6.1.3, BD Biosciences). Compensation was applied to adjust for spectral overlap during multicolor flow cytometry. Analysis of all samples was run in parallel with matched isotype controls at the same concentration as each antibody. Samples were analyzed by first gating on live cells by forward and side scatter. To confirm viability, samples were stained with either Annexin V/PI (Sigma-Aldrich) when surface labeling or Fixable Viability Dye eFluor 660 (eBioscience, San Diego, CA, USA) when intracellular labeling. Viability of samples was routinely greater than 90%. Doublets were excluded from cell cycle analysis of MCF7 cells by gating on pulse height versus pulse area. Immunofluorescence of sorted MSCs was reanalyzed as a quality control of population purity. MSCs were designated as positive for expression of a specific antigen when their immunofluorescence intensity exceeded the 99th percentile of the fluorescence distribution for the isotype control. Histograms of antigen expression were generated by flow cytometric analysis of *n* = 10,000 cells.

### Proliferation and differentiation potential

To determine colony-forming efficiency, MSCs were inoculated at the clonogenic level of 100 ± 10 cells in a 10-cm tissue culture dish with 10 ml CCMA. After 2 weeks in a humidified incubator at 37 °C with 5% CO_2_, MSCs were stained with crystal violet (Sigma-Aldrich) as described by Barrilleaux et al. [34], and resultant cell colonies were visually counted. To evaluate growth kinetics and doubling times, MSCs were inoculated at 100 ± 10 cells/cm^2^ in 24-well plates with 0.5 ml CCMA/well, with complete medium exchange every other day for a total of 8 days. Specific growth rate of the cells was calculated according to Russell et al. [38].

Osteogenesis and adipogenesis were induced in MSC cultures that were ∼ 75% confluent and analyzed after 21 days of differentiation as described by Russell et al. [38]. Alizarin Red S (Sigma-Aldrich) was used to stain calcified extracellular matrix indicative of osteogenesis. AdipoRed reagent (Lonza, Walkersville, MD, USA) detected lipid accumulation during adipogenesis. Spectral absorbance of extracted Alizarin Red S was measured at 562 nm. Fluorescence emission intensity of AdipoRed was measured at 572 nm after excitation at 485 nm. These metrics of differentiation are reported on a per microgram DNA basis. DNA mass was measured with the Quant-iT™ PicoGreen® dsDNA Assay Kit and calibrated with λ DNA as a standard.

### Senescence-associated β-galactosidase (SA β-Gal) activity

SA β-Gal activity was detected in subconfluent MSC cultures inoculated at 1000 cells/cm^2^ into 24-well plates on day 4 after inoculation using the Senescence β-Galactosidase Staining Kit (Cell Signaling Technology). Images of cells stained for SA β-Gal activity were captured with Image-Pro Plus software [39] (version 6.1, Media Cybernetics, Rockville, MD, USA) through an Optronics DEI-750 digital camera (Goleta, CA, USA) mounted on an Olympus IX50 microscope (Center Valley, PA, USA). The percentage of MSCs that stained positive for SA β-Gal activity was evaluated by image analysis of *n* = 300 cells per biological replicate.

### Statistical analysis

Differentiation measurements are reported as standardized scores relative to negative control values by centering and scaling each measurement using the mean and standard deviation for confluent monolayers of MSCs cultured in CCMA without differentiation agents. The central limit theorem for a large sample size was applied to measurements of antigen expression by flow cytometric analysis (*n* =10,000 cells) and SA β-Gal activity by image analysis (*n* = 300 cells). For these data, we employed the following parametric tests for sampled populations that are normally distributed: two-tailed Student’s *t* test to assess differences between two cell groups and analysis of variance (ANOVA) in conjunction with a post-hoc Tukey’s honest significant difference test for differences among three or more groups. Nonparametric statistical analysis was applied to all other data (3 ≤ *n* ≤ 6); specifically: 1) the Mann-Whitney test was employed to evaluate differences between two cell groups; 2) Spearman’s rank correlation coefficients (*r*_*s*_) were calculated to quantify relationships between CD264 surface expression and metrics of stem cell fitness; and 3) regression coefficients for these relationships were estimated by Theil’s and Dietz’s methods [40]. Statistical analysis was executed with Minitab software (version 17, State College, PA), and threshold of significance was 0.05 in this study.

## Results

### CD264 detects variation in morphology and SA β-Gal activity of MSCs from older donors

**Fig. 1:**
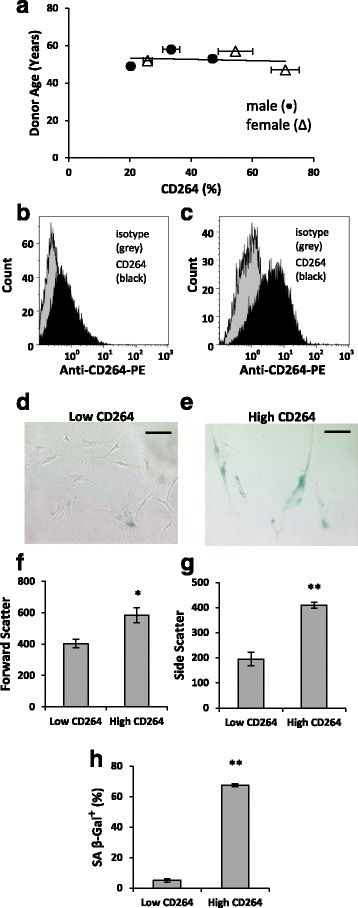
Relationship of CD264 surface expression to morphology and senescence of MSCs from older donors. MSCs were harvested from age-matched male (*circle*) and female (*triangle*) donors, 45–60 years old, and analyzed at passage 7. **a** Percentage of CD264^+^ MSCs surface labeled with anti-CD264 monoclonal antibody conjugated to phycoerythrin (*PE*). **b**,**c** Histograms of representative MSC cultures with low (**b**) and high (**c**) percentage of CD264^+^ cells (*black*) relative to isotype (*grey*) from flow cytometric analysis (*n* = 10,000 cells/group). **d**,**e** Phase-contrast micrographs of representative low (**d**) and high (**e**) CD264 cultures. *Scale bars* = 100 μm. **f**,**g** Scatter properties of the CD264 cell groups. **h** Percentage of cells stained positive for senescence-associated β-galactosidase (*SA β-Gal*) activity in the CD264 groups. Data in panels **a** and **f–h** are reported as mean ± SEM for *n* = 3 biological replicates. Nonparametric linear regression line shown in panel **a** for *n* = 6 donor cultures. *p < 0.05, **p < 0.01, versus low CD264 culture

Figure 1a depicts the variation in the content of CD264^+^ cells in bone marrow MSC cultures from donors, 45 to 60 years old (*n* = 6). The chronological age of male and female donors was matched to within 2 years. Specifically, the older MSC donor group contained males 49, 53, and 58 years old and females 47, 52, and 57 years old. We selected MSCs from older donors at passage 7 because of the possibility that cellular aging may be more prevalent in these cultures. The percentage of CD264^+^ MSCs in culture was independent of chronological age and sex for the older donor group.

To explore the possibility of CD264 as a marker of cellular aging for MSCs, we evaluated the morphology and SA β-Gal activity in MSC cultures from two older donors with low and high content of CD264^+^ cells (Fig. 1b and c). For the latter, CD264^+^ cells accounted for upwards of 75% of the MSCs in culture, whereas this value was approximately three times less for low CD264 cultures. The phase-contrast micrographs revealed small, spindle-shaped cells in low CD264 cultures and numerous enlarged cells in MSC cultures with high content of CD264^+^ cells (Fig. 1d and e). This morphology agrees with the increased forward scatter (*p* < 0.05) and side scatter (*p* < 0.01) from high CD264 cultures, which are indicative of larger and more granular cells (*n* = 3; Fig. 1f and g). Only 5% of MSCs in low CD264 cultures stained positive for SA β-Gal versus more than 60% for high CD264 cultures (*p* < 0.01; *n* = 3; Fig. 1h). The enlarged morphology and elevated SA β-Gal activity in high CD264 cultures is consistent with an aging phenotype. Figure 1 suggests that CD264 is not a biomarker of the chronological age of the MSC donor (Fig. 1a), but instead its surface expression is indicative of the cellular age of the MSC culture (Fig. 1d–h).

It is noteworthy that the surface expression of the standard MSC markers (CD73, CD90, and CD105) was similar in low and high CD264 cultures (Additional file 4: Figure S3). The inability of the standard MSC immunophenotype to detect differences in the content of aging cells in MSC cultures demonstrates the need for a new set of surface markers, such as CD264, to identify specific cell populations in MSC cultures.

### Content of CD264^+^ MSCs inversely correlates to proliferation and differentiation potential

**Fig. 2:**
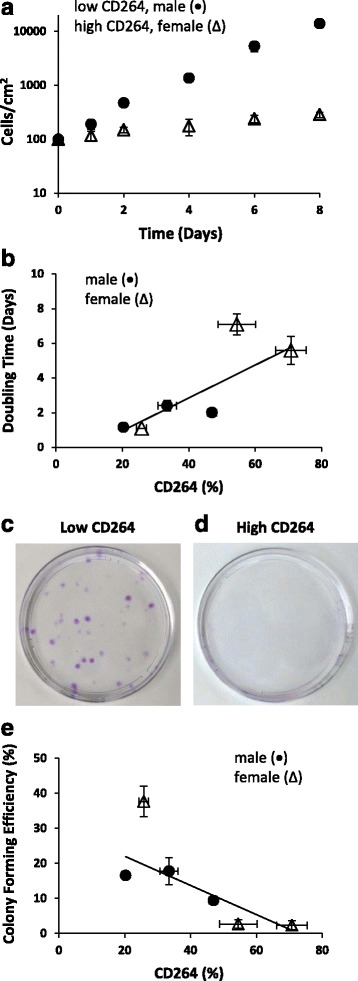
Proliferation potential of older donor MSCs as a function of CD264^+^ cell content. MSCs from male (*circle*) and female (*triangle*) donors ages 45–60 years old are described in the legend to Fig. 1. **a** Growth curve of representative low (*circle*) and high (*triangle*) CD264 cultures. **b** Doubling time of MSC cultures calculated from the growth curve. **c**,**d** Phase-contrast micrographs of stained clonogenic colonies from representative low (**c**) and high (**d**) CD264 cultures in tissue culture Petri dishes (10-cm diameter). **e** Colony-forming efficiency of MSCs. Data are reported as mean ± SEM for *n* = 3 biological replicates. Nonparametric linear regression lines shown in panels **b** and **e** for *n* = 6 donor cultures

**Fig. 3:**
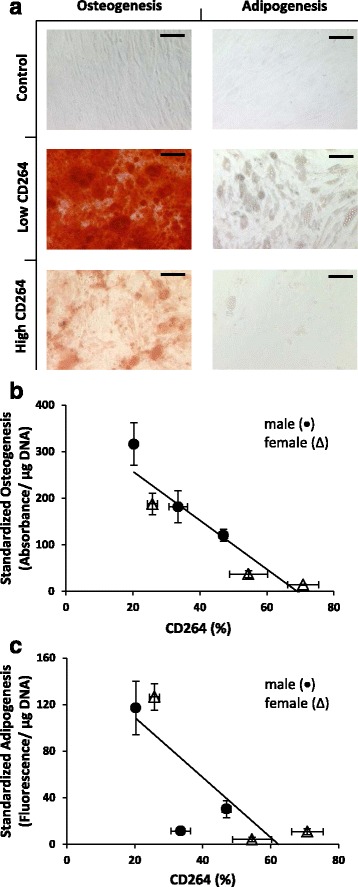
Differentiation potential of older donor MSCs as a function of CD264^+^ cell content. P7 MSCs from older donors, 45–60 years old, were cultured for 21 days in either osteogenic or adipogenic medium. **a** Representative low and high CD264 cultures stained with Alizarin Red S to detect mineralization during osteogenesis and AdipoRed to detect lipid accumulation during adipogenesis. Negative control cultures maintained in growth medium. *Scale bars* = 100 μm. **b**,**c** Absorbance from extracted Alizarin Red S (**b**) and fluorescence from AdipoRed (**c**), respectively, are reported per microgram DNA and standardized relative to the negative control. MSCs were harvested from male (*circle*) and female (*triangle*) donors. Data are reported as mean ± SEM for *n* = 3 biological replicates. Nonparametric linear regression lines in panels **b** and **c** shown for *n* = 6 donor cultures

Among MSC cultures from donors ages 45 to 60 years old, there was no correlation between the chronological age of the donor and established metrics of the proliferation and differentiation potential of in vitro MSC cultures (Additional file 5: Figure S4). Moreover, there were no significant sex-linked differences in the proliferation and differentiation potential of MSCs from age-matched males and females in the older donor group (Figs. 2 and 3). Because chronological age and sex are not indicative of stem cell fitness for older donor MSCs, a different parameter is required to predict the proliferation and differentiation potential of MSCs from this group.

In contrast to chronological age, the content of CD264^+^ cells was inversely related to the proliferation potential of the MSC cultures from donors ages 45–60 years old (Fig. 2). When the low and high CD264 cultures shown in Fig. 1 were expanded ex vivo from an inoculum of 100 cells/cm^2^, the former grew more rapidly to accumulate nearly 50 times more cells after 8 days of proliferation (*n* = 3; Fig. 2a). Cell doubling time was highly correlated to the percentage of CD264^+^ cells in MSC cultures, with an average Spearman’s rank correlation coefficient of 0.8 (*p* < 0.05; *n* = 6; Fig. 2b). Another measure of proliferation potential is the capacity of MSC cultures to form clonogenic colonies in tissue culture dishes. Colony-forming efficiency decreased with higher content of CD264^+^ cells (average *r*_*s*_ = –0.8, *p* < 0.05; *n* = 6; Fig. 2c–e) and was negligible for MSC cultures that contained > 50% CD264^+^ cells (Fig. 2e). The limited proliferation potential of high CD264 cultures (Fig. 2) coupled with their larger cell size and SA β-Gal staining (Fig. 1) are classic features of cellular aging.

There was a similar trend in differentiation potential. After low and high CD264 cultures of MSCs from the older donors in this study were cultivated in osteogenic medium for 3 weeks, Alizarin Red S staining detected extensive calcium deposition during matrix mineralization in low CD264 cultures, whereas the staining was weaker in high CD264 cultures (Fig. 3a). Absorbance from extracted Alizarin Red S is reported on an intrinsic basis per microgram DNA and standardized to a negative control culture maintained in growth medium (Fig. 3b). We detected a strong inverse correlation between this measure of osteogenic potential and the percentage of CD264^+^ cells in MSC cultures from donors ages 45–60 years old (*r*_*s*_ = −1.0, *p* < 0.01; *n* = 6; Fig. 3b). In particular, there was a 10-fold decrease in our standardized score of Alizarin Red S staining as CD264^+^ cell content increased from 20% to 75%. Similar results were obtained for lipid accumulation during adipogenesis (average *r*_*s*_ = −0.8, *p* < 0.05; *n* = 6; Fig. 3c).

**Fig. 4:**
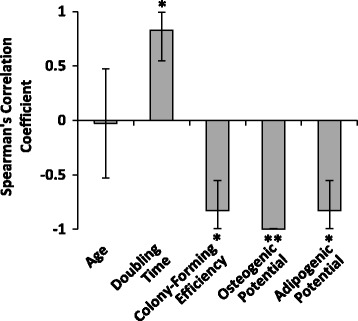
Summary of correlations of CD264^+^ cell content of older donor MSCs to metrics of stem cell fitness. Spearman’s rank correlation coefficients relating the percentage of CD264^+^ cells in MSC cultures to the following parameters: donor age, doubling time, colony-forming efficiency, osteogenic potential, and adipogenic potential as in Figs. 1, 2, and 3. Data are reported as *r*_*s*_ ± SE for *n* = 6 donor cultures

Figure 4 provides a summary of the correlations of CD264^+^ cell content to the metrics of stem cell fitness in this study for MSC cultures from donors ages 45–60 years old. While there is no correlation with the donor’s chronological age, there is a consistently strong correlation (average value of |*r*_*s*_| ≥ 0.8) with each metric of proliferation and differentiation potential. These findings suggest that CD264 may have application as a cell surface marker to predict stem cell fitness of marrow-derived MSCs from older donors.

**Fig. 5:**
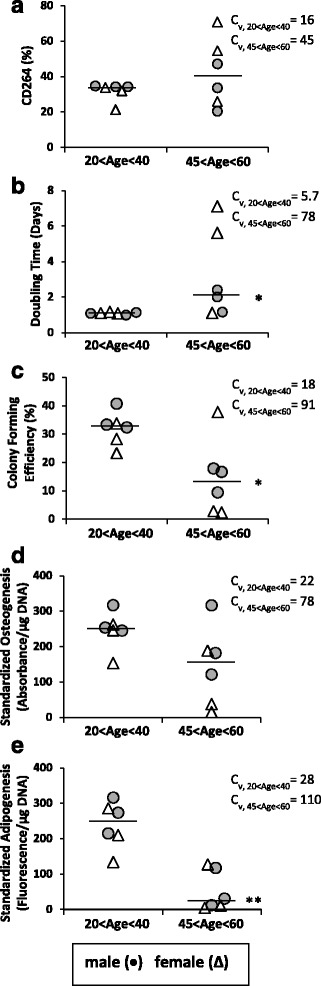
Comparison of CD264 surface expression, proliferation potential, and differentiation potential of MSCs from young and older donors. Individual value plots depict P7 MSCs from age-matched male (*circle*) and female (*triangle*) donors separated into two chronological age groups: 20–40 and 45–60 years old. Parameters measured were percentage of CD264^+^ MSCs (**a**), doubling time (**b**), colony-forming efficiency (**c**), osteogenic potential (**d**), and adipogenic potential (**e**) as in Figs. 2 and 3. Median values are depicted as bars. Coefficient of variation (*C*_*v*_) given for each chronological age group and each measured parameter. Sample sizes: *n* = 3 biological replicates per donor culture; *n* = 6 donor cultures per chronological age group. *p < 0.05, **p < 0.01, versus 20–40 years age group

We extended our study to MSC cultures from young donors. This group of MSC donors contained males ages 24, 29, and 37 years old and females ages 24, 30, and 36 years old. Individual value plots in Fig. 5 compare our results from older donor MSCs (ages 45–60 years, *n* = 6) with those from MSCs at the same passage harvested from donors between 20 and 40 years old (*n* = 6). No significant sex-linked differences were detected in MSCs from age-matched male and female donors in the young donor group. CD264^+^ cells accounted for 20–35% of the MSC culture from young donors (Fig. 5a), and these MSCs exhibited extensive proliferation and differentiation (Fig. 5b–e). For example, the doubling time was nearly once per day (Fig. 5b) and intrinsic differentiation per microgram DNA was a minimum of 100 standard deviations higher than the mean for the negative control (Fig. 5d and e). When our results from young donors in Fig. 5 are taken in tandem with the correlations in Figs. 2 and 3, they suggest a threshold of 20–35% CD264^+^ MSCs above which proliferation and differentiation potential become increasingly compromised with higher CD264^+^ cell content.

Our comparison between the young and older donor groups revealed significant slower doubling time (*p* < 0.05; Fig. 5b), lower colony-forming efficiency (*p* < 0.05; Fig. 5c), and less adipogenic potential (*p* < 0.01; Fig. 5e) for the older donor group (*n* = 6 per group). In addition, there was more donor variation in the percentage of CD264^+^ cells and the four metrics of stem cell fitness for donors ages 45–60 years old. For each graph in Fig. 5, the coefficient of variation for the older donor group exceeded 2.5 times the value for the young group. While we analyzed MSCs from donors that spanned nearly four decades of life, the high degree of variation in the measured parameters for the older donors obscured differences between MSCs from young and older donors in the content of CD264^+^ cells (Fig. 5a) and in the extent of calcium deposited in vitro during osteogenesis (Fig. 5d). These data imply that chronological age of the donor is not a reliable indicator of osteogenic potential, and they reinforce the need for a predictive biomarker of cellular age, such as CD264.

### Sorted CD264^+^ MSCs are enlarged and grow slower than CD264^-^ MSCs

**Fig. 6:**
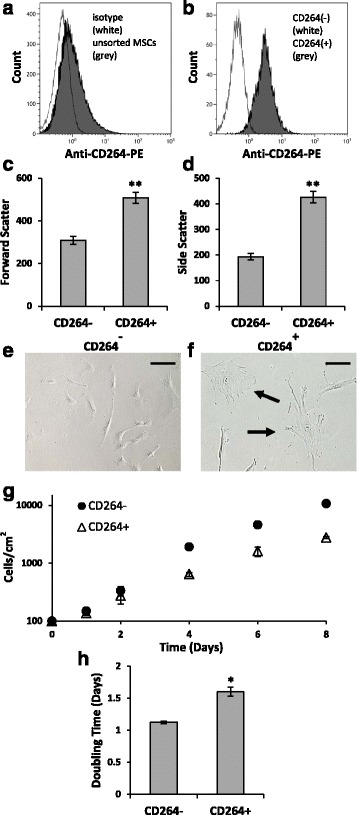
Morphology and growth of sorted CD264^–^ and CD264^+^ MSCs. P6 MSCs from a female donor (age 52 years) were surface labeled with anti-CD264-phycoerythrin (*PE*) and sorted into CD264-negative and CD264-positive groups. **a**,**b** Histograms depict MSCs before (**a**) and after (**b**) cell sorting (*n* = 10,000 cells/group). **c**,**d** Scatter properties of the sorted cell groups. **e**,**f** Representative phase-contrast micrographs of the sorted MSCs. *Arrows* point to enlarged cells with flattened, granular cytoplasm. *Scale bars* = 100 μm. **g**,**h** Growth curve of CD264^–^ (*circle*) and CD264^+^ (*triangle*) cultures (**g**) and corresponding doubling times (**h**). Data are reported as mean ± SEM for *n* = 3 biological replicates. *p < 0.05, **p < 0.01, versus CD264^–^ MSCs

We sorted MSCs from one of the older donors (age 52, female) into CD264-negative and CD264-positive groups (Fig. 6a and b). The scatter properties of the sorted groups indicate that CD264^+^ MSCs were larger (forward scatter) and more granular (side scatter) relative to CD264^–^ MSCs (*p* < 0.01; *n* = 3; Fig. 6c and d). This finding was validated by visual inspection of the sorted cultures. Only CD264^+^ cultures contained enlarged MSCs with a flattened, granular cytoplasm (Fig. 6e and f). Moreover, CD264^+^ cultures grew more slowly during ex vivo expansion. After 8 days of proliferation, CD264^+^ cultures contained fewer cells by a factor of 3–4 (Fig. 6g). This corresponds to an average doubling time of ~ 1.0 day for CD264^–^ MSCs versus > 1.5 days for CD264^+^ MSCs (*p* < 0.05; *n* = 3; Fig. 6h). Results in Fig. 6 from cell sorting are consistent with those in Figs. 1 and 2 from heterogeneous MSC cultures in that both data sets support CD264 as a marker to identify MSCs with two characteristic traits of cellular aging: larger size and slower growth.

### CD264 has a similar expression profile to that of p21 during serial passage of MSCs

**Fig. 7:**
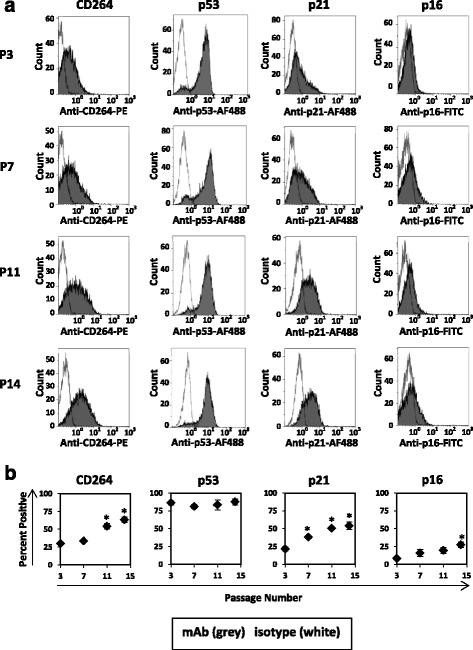
Expression of CD264 and cell cycle inhibitors in MSCs during serial passage. MSCs from a 36-year-old female donor were labeled on the cell surface with anti-CD264-phycoerythrin (*PE*), fixed, and permeabilized. Cell cycle inhibitors were detected by labeling fixed and permeabilized MSCs with anti-p53-Alexa Fluor (*AF*)488, anti-p21-AF488, and anti-p16-fluorescein isothiocyanate (*FITC*). **a** Representative histograms from flow cytometric analysis of single-labeled MSCs with monoclonal antibodies (*mAbs*) against CD264 and cell cycle inhibitors (*grey*) or isotype (*white*) at select passages (*P*; *n* = 10,000 cells/group). **b** Percentage of MSCs positive for CD264, p53, p21, and p16 as a function of passage number (*n* = 3 biological replicates). Cumulative cell doublings during serial passage: 15–17 (P3), 24–27 (P7), 31–33 (P11), and 35–38 (P14). Data are reported as mean ± SEM. *p < 0.05 versus P3 MSCs

To determine the stage of cellular aging in which CD264 is upregulated, we compared the temporal profile for CD264 expression in MSCs to that of select cell cycle inhibitors during serial passage. We employed MSCs from a 36-year-old donor. More than 70% of the MSCs were positive for p53 throughout serial passage (Fig. 7a and b). In contrast, there was a distinct shift in the histograms for CD264 and p21 towards higher fluorescence during serial passage (Fig. 7a). The percentage of CD264^+^ MSCs doubled between P3 and P11, and there was a similar increase for p21^+^ cells (*p* < 0.05; *n* = 3; Fig. 7b). We detected p16 expression in < 35% of MSCs during serial passage (Fig. 7a and b), which is in sharp contrast to the strong expression of this antigen on positive control cells (Additional file 2: Figure S1). Our study reveals that CD264 is upregulated after p53 and has a similar expression profile to that of p21 during serial passage.

CD264 surface expression was measured during serial passage of MSCs from two additional donors: a male age 29 years, and a female age 52 years. Consistent with our other data, CD264 expression was a function of passage number but independent of the chronological age and sex of the donor. For both MSC cultures, the temporal profile for CD264 expression was similar to that in Fig. 7; the percentage of CD264^+^ cells was in the mid 20s to mid 30s from passage 3–7 and then increased to upwards of 90% by passage 14 (*p* < 0.05; *n* = 3; Additional file 6: Figure S5). These findings are supportive of CD264 as a surface marker of cellular aging induced by replicative stress during serial passage of MSCs.

**Fig. 8:**
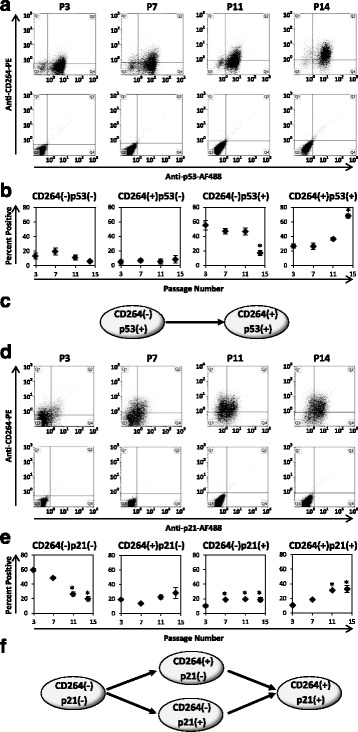
Co-expression of CD264 with p53 and p21 in MSCs during serial passage. MSCs corresponding to Fig. 6 were surface labeled with anti-CD264-phycoerythrin (*PE*), fixed, permeabilized, and co-labeled with either anti-p53-Alexa Fluor (*AF*)488 (**a**,**b**) or anti-p21-AF488 (**d**,**e**) monoclonal antibodies. **a**,**d** Representative bivariate histograms of co-labeled MSCs at select passages (*n* = 10,000 cells/group). **b**,**e** Corresponding percentages of MSCs from each quadrant of the bivariate histograms (*n* = 3 biological replicates). **c**,**f** Schematic of the pattern of expression of CD264 with p53 (**c**) and p21 (**f**) during serial passage. Donor age 36 years old. Data are reported as mean ± SEM. *p < 0.05, versus passage (*P*)3 MSCs

We examined co-expression of CD264 with p53 and p21. MSCs progressed from a CD264^–^p53^+^ phenotype at early passage to a CD264^+^p53^+^ phenotype at late passage (Fig. 8a–c). Throughout serial passage, nearly all the CD264^+^ MSCs co-expressed p53, with the CD264^+^p53^–^ fraction accounting for 10% or less of the MSCs in culture (Fig. 8b). CD264 and p21 had a more complex pattern of expression. The majority of MSCs exhibited a CD264^–^p21^–^ phenotype at early passage (Fig. 8d and e). The percentage of CD264^–^p21^–^ cells decreased by a factor of three during serial passage as they progressed to one of two transitional states: CD264^+^p21^–^ or CD264^–^p21^+^ (*p* < 0.05; *n* = 3; Fig. 8e). These two intermediate cell fractions converted to a CD264^+^p21^+^ phenotype, which accumulated at P11 and P14 (Fig. 8d and e). The pattern of expression for CD264 and p21 is summarized in Fig. 8f. The schematic depicts the progression of MSCs from a CD264^–^p21^–^ phenotype at early passage through transitional states to a CD264^+^p21^+^ phenotype at late passage.

## Discussion

### CD264: a surface marker of MSC cellular aging

This study demonstrates the potential utility of CD264 as a cell surface marker of cellular aging for heterogeneous MSC cultures derived from human bone marrow. At the start of this project, there had been no studies of donor-to-donor variation of CD264 expression for any type of stem cell. Our analysis revealed that MSC cultures from young (20–40 years) and older (45–60 years) donors exhibited rapid proliferation and extensive differentiation when 20% to 35% of the cells in culture were positive for CD264. MSC cultures from older donors within this range of CD264^+^ cell content were small in size and had negligible SA β-Gal activity despite the advanced chronological age of the donor. Published histograms indicate a similar composition of CD264^+^ cells in early passaged MSC cultures [29]. A possible reason for a basal level of CD264 expression in MSCs is that ex vivo expansion of these cultures has caused some degree of cellular aging from DNA damage due to rapid cell division [41], but not enough to significantly curtail stem cell fitness. Another possibility for this basal level is that CD264 may participate in cellular functions unrelated to senescence, as suggested by its expression in a wide variety of human fetal and adult tissues [25, 26].

When CD264^+^ cell content exceeded the 35% threshold in our MSC cultures, CD264 surface expression was inversely correlated to the proliferation and differentiation potential of the culture. All metrics of stem cell fitness in this study were severely compromised in MSC cultures in which 75% of the cells were positive for CD264 surface expression. At this high level of CD264^+^ cell content, the majority of MSCs in culture were enlarged and senescent. The slower growth and larger size of CD264^+^ MSCs were validated by cell sorting. These findings are consistent with a report of increased expression of CD264 expression in late passage, senescent MSCs [31].

Upregulation of the decoy TRAIL receptor CD264 in MSCs from older donors and during serial passage is possibly an attempt by the cells to evade senescence. In addition to initiating the apoptosis cascade, TRAIL stimulates cell survival and proliferation in some circumstances [42]. Due to its truncated death domain, CD264 is incapable of propagating the apoptosis signal from TRAIL [22]; however, it retains the ability to mediate non-apoptotic TRAIL signaling [43]. MSCs may increase CD264 expression as a means to defend against cellular senescence by promoting cell survival and growth.

It is noteworthy that chronological age of our MSC donors was not a reliable metric of stem cell fitness. Consistent with previous reports [44, 45], we detected substantial variation in the proliferation and differentiation potential of MSC cultures from older donors (45–60 years) at the same passage, even between age-matched donors. While CD264 expression was inversely correlated to stem cell fitness, its expression was independent of the number of years our older MSC donors had lived. These results are supportive of CD264 as a marker of the cellular age of MSCs, not the chronological age of the donor. Differences between the chronological age of an organism and cellular age of its tissues can arise in high-turnover tissues when cells replicate regularly, leading to an accumulation of DNA damage [46]. Cellular aging of stem cells can be accelerated; for example, by replicative stress during the repair of damaged tissue from injury and disease.

### Link of CD264 with p53 and p21

In the literature, comparisons of dividing to senescent cells have suggested possible links of CD264 to cell cycle inhibitors p16, p21, and p53 [23, 24, 31]; however, these cell cycle inhibitors are upregulated at very different stages of cellular aging [47, 48]. One possible reason for these inconsistencies is that the stage of cellular aging at which CD264 is upregulated is dependent on cell type. Alternatively, comparisons of early passage to late passage cells without intermediate time points may have led to spurious links between CD264 and cell cycle inhibitors in the earlier studies.

To address these inconsistencies, we investigated the temporal expression of CD264 relative to the aforementioned cell cycle inhibitors during serial passage of MSCs. Our cultures were positive for p53 at early passage consistent with previous reports for bone marrow MSCs [19, 48]. Expression of p53 in early passage MSCs may be in response to DNA damage accumulated in MSCs during ex vivo expansion or the life of the donor [49]. Alternatively, p53 expression at early passage may reflect its role in regulating the proliferation and differentiation of MSCs [50, 51]. We observed that CD264 was upregulated after p53. In our study, nearly all of the CD264^+^ MSCs co-expressed p53. These two observations are consistent with the scenario that the *DcR2* gene encoding CD264 could be a p53-target gene in marrow MSCs, as is the case for multiple tumor cell lines [52, 53]. The p53 binding site is located in the first intron of the *DcR2* gene [53].

In our time-course study, CD264 and p21 had the most similar expression profiles; both were upregulated between passages 7 and 11, at an intermediate stage of cellular aging. We observed that co-expression of p21 and CD264 was achieved after MSCs passed through CD264^+^p21^–^ and CD264^–^p21^+^ transitional states, suggesting that perhaps p21 and CD264 could be upregulated by different regulatory factors during the cellular aging of MSCs. This is possible given that the *p21* gene exhibits both p53-dependent and p53-independent activation [54].

We observed that CD264 was upregulated before a significant increase in p16 expression. Upregulation of p16 is a key event in the terminal phase of cell cycle arrest and senescence [55]. Earlier studies report CD264 as a marker of senescence for various cell types [23, 24, 56]. The temporal order of CD264 and p16 expression in our experiment implies that CD264 does not appear to be strictly a marker of senescence as previously thought. Instead, our data suggest that CD264 is first upregulated in MSCs at an intermediate stage of cellular aging and remains upregulated as aging progresses towards senescence.

### Applications

The ability of CD264 to detect cellular aging in MSCs has several applications. We envision that CD264 expression could be used as a metric to rapidly screen the cellular age of MSC preparations from older patients because chronological age is not a reliable measure of stem cell fitness, as demonstrated in this study. Low CD264 expression would be an indicator that the patient could be a potential candidate for autologous MSC transplantation, such as for bone repair.

CD264 could be an effective indicator to select culture conditions for ex vivo expansion of MSCs. Addition of zoledronate, a bisphosphate, to culture media limits accumulation of DNA damage in MSCs during ex vivo expansion and preserves their proliferation and differentiation potential by inhibiting mTOR signaling [17]. Monitoring CD264^+^ cell content in MSC cultures could expedite the selection of culture conditions to slow the cellular aging of MSCs.

Standardizing the composition of heterogeneous MSC cultures by negative selection with CD264 could improve their regenerative potential. When preparing MSC therapies, CD264^+^ cells would probably account for a quarter or more of MSCs from young and older donors, as they did in our study. Currently, negative selection of marrow aspirates with hematopoietic surface markers, such as CD45 and glycophorin A, depletes hematopoietic cells and enriches for colony-forming MSCs [16]. CD264 could be added to this cocktail of surface markers to manufacture MSC therapies with reliable efficacy.

Enrichment of CD264^+^ cells could prove advantageous for exploring the biology of cellular aging by resolving conflicting data in the literature about the age-dependent decline in the regenerative potential of MSCs. As an example, osteogenic differentiation has been reported to either decrease with the donor’s chronological age or remain unchanged for both old and young donors [57, 58]. Standardizing the composition of heterogeneous MSC cultures with CD264 by positive selection could yield more consistent results.

Positive selection with CD264 could allow for rejuvenation of aging MSCs to partially restore their proliferation and differentiation potential. Existing rejuvenation strategies have used, for instance, transient p38 inhibition to improve the regenerative capacity of aging skeletal muscle stem cells [18]. A similar strategy could be effective in restoring the regenerative properties of CD264^+^ MSCs. Results from our time-course experiment are favorable for the potential rejuvenation of CD264^+^ MSCs. There could be an optimal stage of cellular aging to restore cell function. For instance, targeting p53 and the retinoblastoma protein reversed senescence in fibroblasts and epithelial cells with low levels of p16 expression, but they failed to restore growth when p16 expression was high [59]. Similarly, rapamycin restored growth of fibrosarcoma cells 3 days after the onset of senescence, but it had only a minimal improvement after the cells were senescent for 9 days [60]. The low level of p16 expression in CD264^+^ MSCs until late passage presents a time window when their rejuvenation could be feasible because the cells are at an intermediate stage of cellular aging.

## Conclusions

This study establishes CD264 as the first cell surface marker of cellular aging for human bone marrow MSCs. There are strong, inverse correlations between CD264 expression and metrics of proliferation and differentiation potential. We envision its use to assess patient MSCs for autologous transplantation, to standardize and monitor cell composition during the manufacturing of MSC therapies, and for research to slow and reverse MSC aging. CD264 is a promising tool for clinicians and researchers alike to standardize the composition of heterogeneous MSC cultures and, in so doing, create more efficacious therapies.

## Additional files

Additional file 1: Table S1. Flow cytometric analysis of MSC immunophenotype (PDF 68 kb)

Additional file 2: Figure S1. Positive controls for expression of CD264, p53, and p16 (PDF 94 kb)

Additional file 3: Figure S2. Positive control for expression of p21 (PDF 122 kb)

Additional file 4: Figure S3. Immunophenotype of MSC cultures from older donors with low and high CD264 surface expression (PDF 113 kb)

Additional file 5: Figure S4. Metrics of stem cell fitness for older donor MSCs as a function of donor age (PDF 80 kb)

Additional file 6: Figure S5. Expression of CD264 during serial passage of MSCs from a male (age 29 years) and female (age 52 years) donor. (PDF 153 kb)

## Abbreviations

ATCC: American type culture collection
CCMA: Complete culture medium
DcR2: Decoy TRAIL receptor CD264
ECACC: European collection of authenticated cell cultures
MSC: Mesenchymal stem cell
P: Passage
PI: Propidium iodide
*r*_*s*_: Spearman’s rank correlation coefficient
SA β-Gal: Senescence-associated β-galactosidase
TNFRSF10D: Decoy TRAIL receptor CD264
TRAIL: Tumor necrosis factor-related apoptosis-inducing ligand
TRAIL-R4: Decoy TRAIL receptor CD264
TRUNDD: Decoy TRAIL receptor CD264
