# Supplementary material for: Decoy TRAIL receptor CD264: a cell surface marker of cellular aging for human bone marrow-derived mesenchymal stem cells
Source: Stem Cell Res Ther. 2017 Sep 29;8:201. doi: 10.1186/s13287-017-0649-4 (PMC5622446; doi:10.1186/s13287-017-0649-4)
Supplement: Supplementary file 1 — Flow cytometric analysis of MSC immunophenotype (PDF 68 kb) [file 13287_2017_649_MOESM1_ESM.pdf]

**Table S1. Flow Cytometric Analysis of MSC Immunophenotype**

| | Antibody[1] | | |
|---|---|---|---|
| **Antigen** | **Fluorochrome** | **Supplier** | **MSC Expression[2]** |
| CD11b | PC5 | Beckman Coulter | -[3] |
| CD19 | ECD | Beckman Coulter | - |
| CD79α | PC5 | Beckman Coulter | - |
| CD34 | PE | Beckman Coulter | - |
| CD45 | PC7 | Beckman Coulter | - |
| HLA- DR | FITC | BD Biosciences | - |
| CD73 | PE | eBioscience | + |
| CD90 | FITC | Beckman Coulter | + |
| CD105 | PE | Beckman Coulter | + |

[1]Fluorochrome-conjugated, anti-human monoclonal antibodies were used to immunolabel MSCs.

[2]All MSC donor preparations employed in our project exhibited the immunophenotype depicted in this table.

[3]Notation: (-) $\leq$ 2% of the MSCs expressed the antigen; (+) $\geq$ 95% of the MSCs were positive for antigen expression.
