# Supplementary material for: Decoy TRAIL receptor CD264: a cell surface marker of cellular aging for human bone marrow-derived mesenchymal stem cells
Source: Stem Cell Res Ther. 2017 Sep 29;8:201. doi: 10.1186/s13287-017-0649-4 (PMC5622446; doi:10.1186/s13287-017-0649-4)
Supplement: Supplementary file 2 — Positive controls for expression of CD264, p53, and p16 (PDF 94 kb) [file 13287_2017_649_MOESM2_ESM.pdf]

Corresponding Author: Kim O'Connor

Figure S1

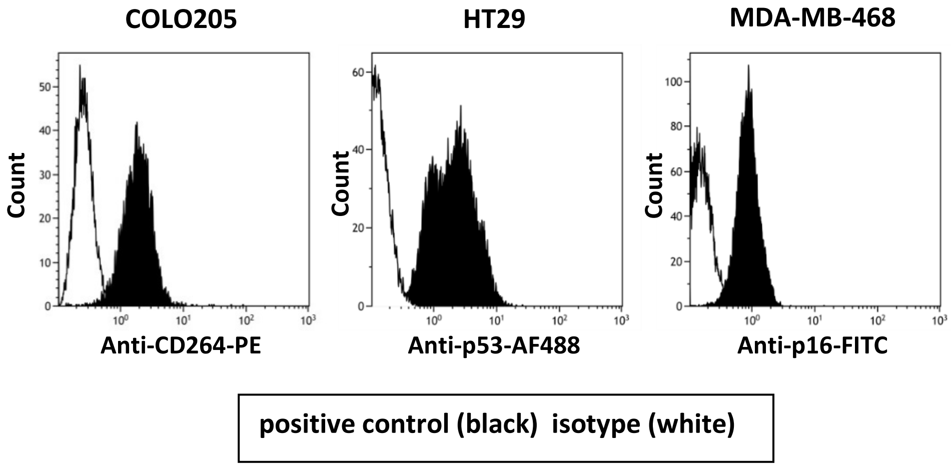

Fig. S1 Positive controls for expression of CD264, p53 and p16. Flow cytometric analysis of CD264 expression on COLO 205 human colon cancer cells, p53 expression on HT29 human colon cancer cells, and p16 expression on MDA-MB-468 human breast cancer cells. For intracellular antigens, control cells were fixed and permeabilized with formaldehyde/methanol for labeling p16 and BD Biosciences Transcription Factor Buffer Set for p53. Histograms from positive controls (black) and isotypes (white). Sample size: n = 10,000 cells/group.
