# Supplementary material for: Decoy TRAIL receptor CD264: a cell surface marker of cellular aging for human bone marrow-derived mesenchymal stem cells
Source: Stem Cell Res Ther. 2017 Sep 29;8:201. doi: 10.1186/s13287-017-0649-4 (PMC5622446; doi:10.1186/s13287-017-0649-4)
Supplement: Supplementary file 3 — Positive control for expression of p21 (PDF 122 kb) [file 13287_2017_649_MOESM3_ESM.pdf]

Figure S2

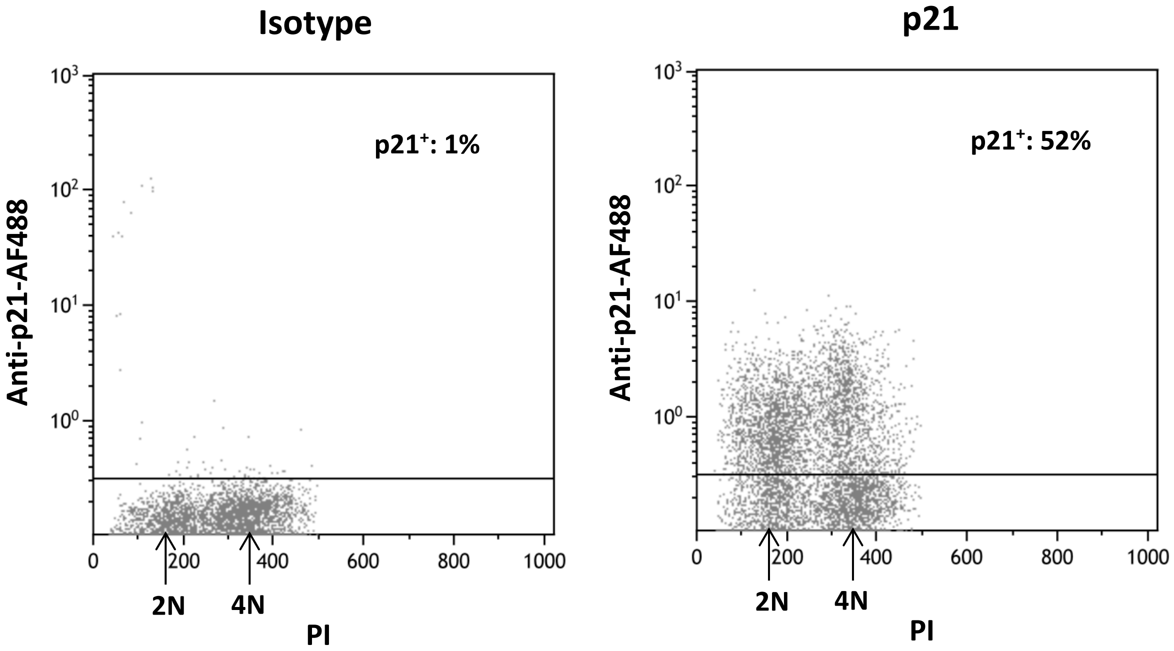

Fig. S2 Positive control for expression of p21. Bivariate analysis of p21 expression versus PI staining of DNA for MCF7 human breast cancer cells exposed to 50 nM Taxol for 36 h. Cells samples were gated on pulse height versus pulse area to exclude doublets. MCF7 cells were fixed with formaldehyde and permeabilized with methanol before immunolabeling p21 and PI staining of DNA. Histograms show cells with diploid (2N) and tetraploid (4N) DNA content. Sample size: n = 10,000 cells/group.
