# Supplementary material for: Decoy TRAIL receptor CD264: a cell surface marker of cellular aging for human bone marrow-derived mesenchymal stem cells
Source: Stem Cell Res Ther. 2017 Sep 29;8:201. doi: 10.1186/s13287-017-0649-4 (PMC5622446; doi:10.1186/s13287-017-0649-4)
Supplement: Supplementary file 4 — Immunophenotype of MSC cultures from older donors with low and high CD264 surface expression (PDF 113 kb) [file 13287_2017_649_MOESM4_ESM.pdf]

Corresponding Author: Kim O'Connor

Figure S3

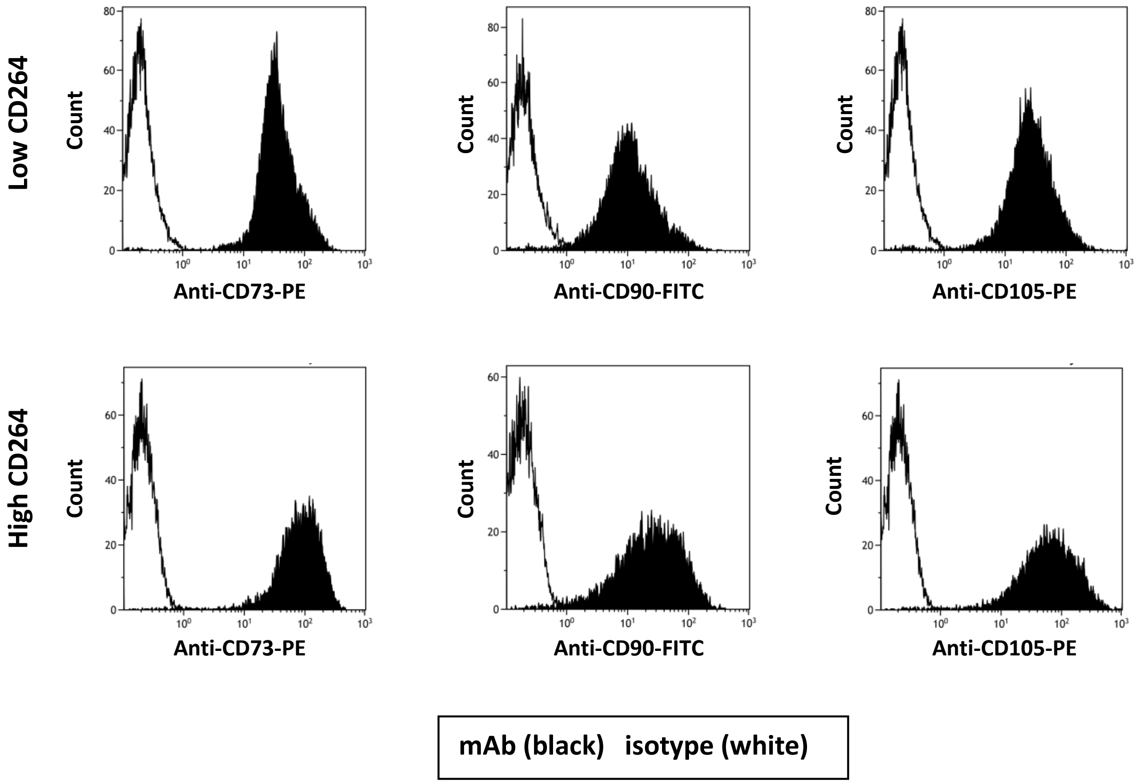

Fig. S3 Immunophenotype of MSC cultures from older donors with low and high CD264 surface expression. Histograms from MSCs labeled with mAbs against CD73, CD90 and CD105 (black) or isotype (white). Donor age: 45-60 years old. Sample size: n = 10,000 cells/group.
