# Supplementary material for: Decoy TRAIL receptor CD264: a cell surface marker of cellular aging for human bone marrow-derived mesenchymal stem cells
Source: Stem Cell Res Ther. 2017 Sep 29;8:201. doi: 10.1186/s13287-017-0649-4 (PMC5622446; doi:10.1186/s13287-017-0649-4)
Supplement: Supplementary file 5 — Metrics of stem cell fitness for older donor MSCs as a function of donor age (PDF 80 kb) [file 13287_2017_649_MOESM5_ESM.pdf]

Figure S4

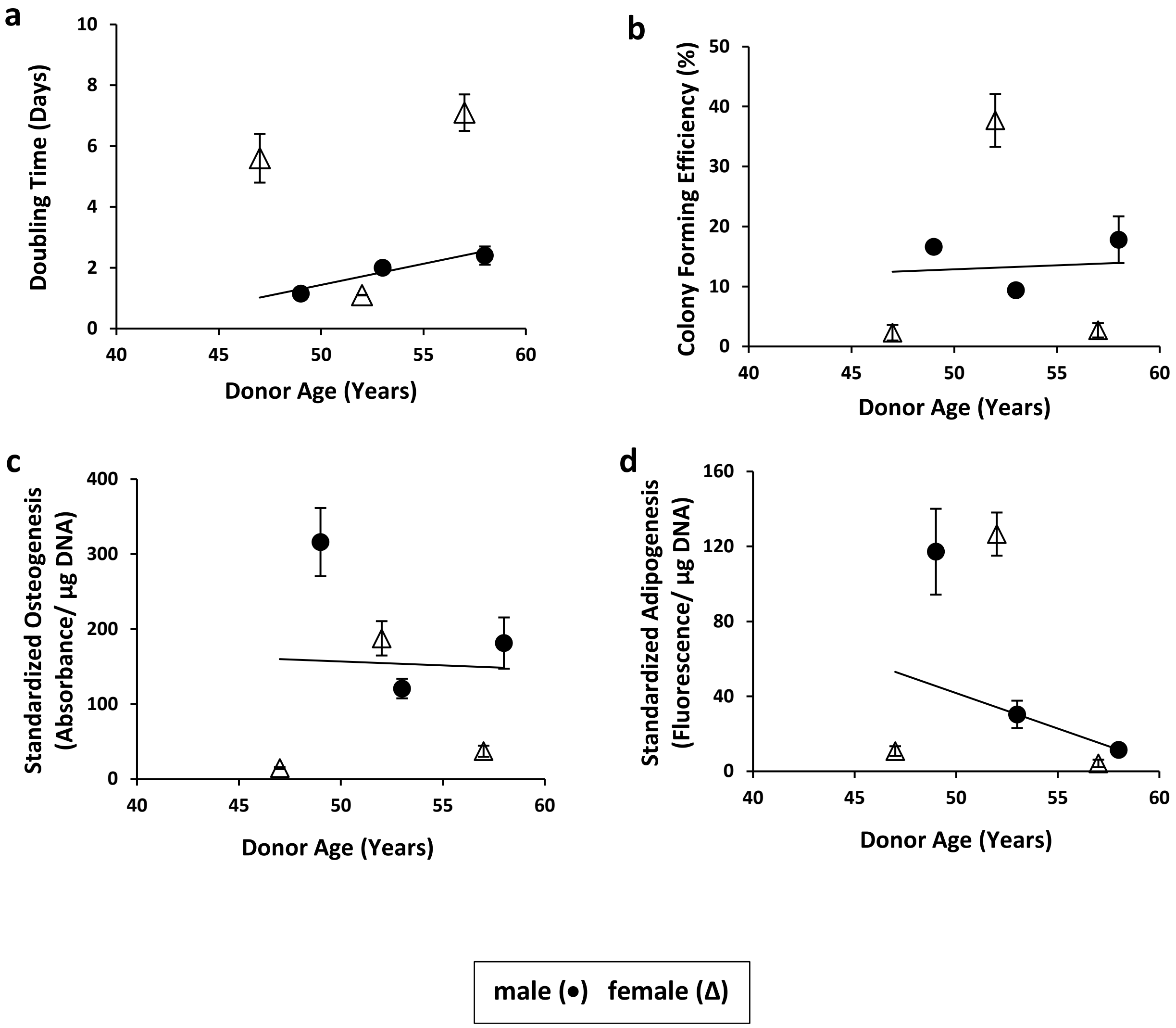

Fig. S4 Metrics of stem cell fitness for older donor MSCs as a function of donor's chronological age. MSCs from male (circle) and female (triangle) donors as in Figure 1. Parameters measured: doubling time (**a**), colony-forming efficiency (**b**), osteogenic potential (**c**) and adipogenic potential (**d**) as in Figures 2 and 3. Data reported as mean ± SEM for n = 3 biological replicates per donor culture. Nonparametric linear regression lines shown for n = 6 donor cultures.
