# Supplementary material for: Decoy TRAIL receptor CD264: a cell surface marker of cellular aging for human bone marrow-derived mesenchymal stem cells
Source: Stem Cell Res Ther. 2017 Sep 29;8:201. doi: 10.1186/s13287-017-0649-4 (PMC5622446; doi:10.1186/s13287-017-0649-4)
Supplement: Supplementary file 6 — Expression of CD264 during serial passage of MSCs from a male (age 29 years) and female (age 52 years) donor. (PDF 153 kb) [file 13287_2017_649_MOESM6_ESM.pdf]

Corresponding Author: Kim O'Connor

Figure S5

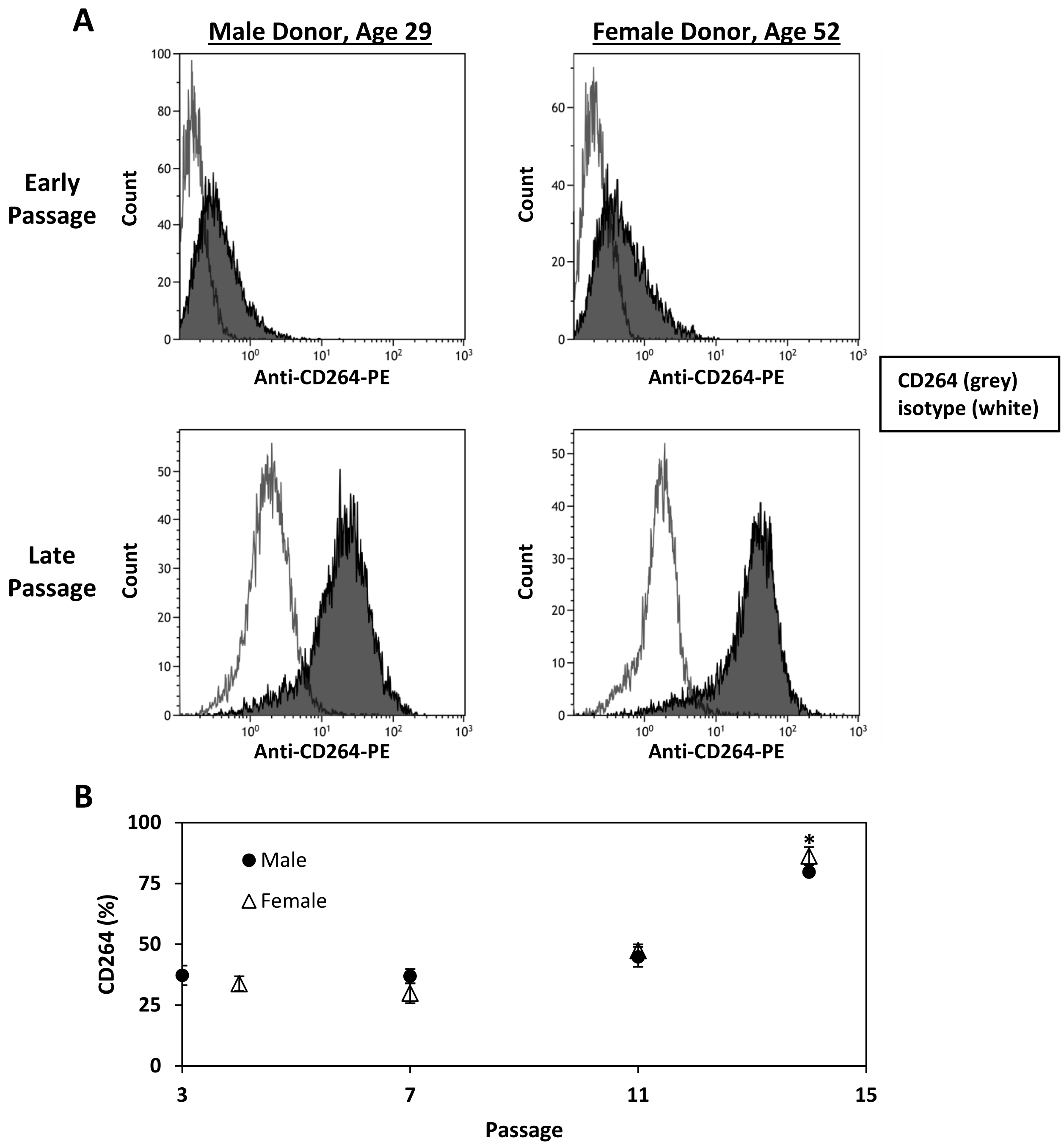

Fig. S5 Expression of CD264 during serial passage of MSCs from a male (age 29) and female (age 52) donor. MSCs were surface labeled with anti-CD264 mAb (grey) or isotype (white). **a** Representative histograms from flow cytometric analysis of labeled MSCs at early and late passage (n = 10,000 cells/group). **b** Percentage of MSCs positive for CD264 as a function of passage number (n = 3 biological replicates). Symbols: (●) male; (∆) female. Data reported as mean ± SEM. *$p < 0.05$ vs. P3/4 MSCs.
